# Electrophysiological characteristics of Chagas disease

**DOI:** 10.1590/S1679-45082013000300006

**Published:** 2013

**Authors:** Swellen Schuenemann Cedraz, Paulo Christo Coutinho da Silva, Ricardo Katsumi Yendo Minowa, Juliano Furtado de Aragão, Danilo Victor Silva, Carlos Morillo, Dalmo Antonio Ribeiro Moreira, Ricardo Garbe Habib, Bruno Pereira Valdigem, Luciana Vidal Armaganijan

**Affiliations:** 1 Instituto Dante Pazzanese de Cardiologia, São Paulo, SP, Brazil

**Keywords:** Chagas disease, Cardiac electrophysiology, Arrhythmias, cardiac, Electrocardiography, Stroke volume

## Abstract

**Objective:**

Chagas disease has become a global problem due to changing migration patterns. An electrophysiological study is generally indicated for assessing sinus node function, conduction through the atrioventricular node and His-Purkinje system, in addition to evaluating the mechanisms of arrhythmia. The aim of this study was to describe the characteristics of electrophysiological study findings in patients with Chagas disease.

**Methods:**

A retrospective descriptive study of 115 consecutive patients with Chagas disease undergoing an electrophysiological study over the last three years in a tertiary hospital in Brazil. Baseline characteristics, electrocardiogram, echocardiogram, and 24-hour Holter monitoring findings were recorded and correlated with the electrophysiological study findings.

**Results:**

The corrected sinus node recovery time and sinoatrial conduction time were abnormal in 6.9% and 26.1% of patients, respectively. Thirty-seven (32.2%) had abnormal atrioventricular conduction. Intraventricular conduction was abnormal in 39 (33.9%). Approximately 48% had induced sustained ventricular arrhythmias, most of which were monomorphic (83.6%). Right bundle branch block was the most common morphology (52.7%). Fifty-one percent were associated with symptoms/hemodynamic instability, 60% required electrical cardioversion, and 27.3% needed overdrive suppression. The most common site of origin was the left ventricular inferoseptal wall (18.2%), followed by the left ventricular posterobasal wall (11%). Patients with an ejection fraction<40% had a 1.94-fold increased risk of ventricular arrhythmias compared to those with an ejection fraction>60% (OR: 1.94; 95%CI: 1.12-3.38; p=0.01). The presence of complex ventricular arrhythmias on Holter did not predict inducible ventricular arrhythmias.

**Conclusions:**

Chagas patients with a low ejection fraction have an increased risk of inducible ventricular arrhythmias. Sinus node dysfunction, and atrioventricular node and His-Purkinje conduction abnormalities occur in about one-third of patients. Complex ventricular arrhythmias on Holter were not associated with an increased risk of inducible ventricular arrhythmias.

## RESUMO

**Objetivo:**

A doença de Chagas constitui importante problema de saúde pública global devido às mudanças nos padrões migratórios. O estudo eletrofisiológico é usualmente indicado na avaliação da função do nó sinusal, condução pelo nó atrioventricular e sistema His-Purkinje e mecanismos das arritmias. O objetivo deste estudo foi descrever as características do estudo eletrofisiológico em pacientes com doença de Chagas.

**Métodos:**

Estudo retrospectivo e descritivo de 115 pacientes consecutivos com doença de Chagas submetidos ao estudo eletrofisiológico nos últimos 3 anos em centro terciário no Brasil. Características basais, eletrocardiográficas, ecocardiográficas e de Holter de 24 horas foram avaliadas e correlacionadas aos achados do estudo eletrofisiológico.

**Resultados:**

Os tempos corrigidos de recuperação do nó sinusal e condução sinoatrial foram anormais em 6,9% e 26,1% dos pacientes, respectivamente. Apresentaram condução atrioventricular anormal 37 (32,2%) pacientes. A condução intraventricular mostrouse alterada em 39 (33,9%) pacientes. Em aproximadamente 48%, houve indução de arritmias ventriculares sustentadas, sendo a maioria monomórfica (83,6%). A morfologia de bloqueio de ramo direito foi a mais comumente observada (52,7%). Dentre as arritmias, 51% associaramse a sintomas/instabilidade hemodinâmica, 60% necessitaram de cardioversão elétrica e 27,3% de estimulação rápida. O sítio de origem mais comum foi a parede inferosseptal do ventrículo esquerdo (18,2%), seguido pela parede posterobasal (11%). Pacientes com fração de ejeção<40% tiveram risco 1,94 vez maior de indução de arritmias ventriculares comparados àqueles com fração de ejeção>60% (OR: 1,94; IC95%: 1,12-3,38; p=0,01). A presença de arritmias ventriculares complexas no Holter não foi preditiva de indução de arritmias ventriculares.

**Conclusões:**

Chagásicos com fração de ejeção baixa apresentam maior risco de arritmias ventriculares induzidas. Disfunção do nó sinusal e anormalidades da condução atrioventricular e do sistema His-Purkinje ocorrem em aproximadamente um terço dos pacientes. Arritmias ventriculares complexas no Holter não foram preditoras de indução de arritmias ventriculares nessa amostra populacional.

## INTRODUCTION

Chagas disease (CD) has become a global problem due to changing migration patterns^(1,2)^. Approximately 13 million people are affected by CD both in endemic and non-endemic countries^(3)^. Approximately 20 to 30% of patients with CD in the so-called indeterminate stage will progress to over cardiomyopathy and develop arrhythmic and/or cardiomyopathic complications, manifested clinically by palpitations, dizziness, syncope, heart failure, and sudden death^(4,5)^. Chronic inflammation usually triggered by parasite persistence may lead to fibrosis and result in sinus node dysfunction, atrioventricular and intraventricular conduction abnormalities, and ventricular tachyarrhythmias due to reentry^(6,7)^. The adequate assessment of patients with CD involves the use of supplementary methods to investigate atrial and ventricular arrhythmias^(5)^. Twelve-lead electrocardiogram (ECG), echocardiogram, and 24-hour Holter monitoring are usually used for risk stratification^(8)^. Electrophysiological testing is generally indicated for the assessment of sinus node function, conduction through the atrioventricular node and His-Purkinje system, and evaluation of the mechanisms of arrhythmia.

## OBJECTIVE

The aim of this study was to describe the characteristics of the electrophysiological findings in patients with Chagas disease.

## METHODS

### Study design

This is a retrospective descriptive study that included all consecutive patients with CD undergoing electrophysiological study (EPS) over the last 3 years in a tertiary hospital in Brazil. Indications for EPS included syncope in 52.2% of cases (two patients with documented sustained ventricular tachycardia), and non-sustained ventricular arrhythmias (frequent premature ventricular contractions and/or non-sustained ventricular tachycardia on Holter monitoring), with or without left ventricular systolic dysfunction in 47.8% of cases. Baseline characteristics (age, hyperlipidemia, history of syncope, tobacco use, prior stroke or transient ischemic attack, systemic arterial hypertension, coronary artery disease, *diabetes mellitus, New York Heart Association* - NYHA - functional class, and concomitant medications), electrocardiogram findings (rhythm, presence of atrioventricular or intraventricular blocks), and echocardiogram findings (ejection fraction, left atrium size, diastolic dysfunction, right ventricle dysfunction, left ventricle thrombus, and left ventricle aneurysm), and 24-hour Holter monitoring findings (premature ventricular contractions, and non-sustained or sustained ventricular tachycardia) were obtained through a chart review, recorded on specifically designed case report forms and correlated with the EPS findings.

This study was approved by the Research Ethics Committee of *Instituto Dante Pazzanese de Cardiologia* (protocol number 4,208). A statistician analyzed the data.

### Study population

All patients with at least two positive serologies for *Trypanosoma cruzi* (ELISA, indirect or direct immunofluorescence) who underwent EPS over the last 3 years were included in the study. Patients with cardiomyopathy secondary to other etiologies such as ischemia (severe coronary artery disease or history of myocardial infarction) and hypertension were excluded.

### Definitions

Low QRS voltage was defined by QRS complex amplitude <0.5mV in frontal leads and <1.0mV in precordial leads, according to the guidelines of the Brazilian Society of Cardiology and Emission Analysis of Electrocardiographic Reports, in 2009^(9)^. Non-sustained ventricular tachycardia was defined as the presence of at least three consecutive ventricular beats at a heart rate of >100bpm. Sustained ventricular tachycardia was defined as tachycardia originating in the ventricles with a heart rate of at least 100bpm, lasting at least 30 seconds and/or associated with hemodynamic or clinical instability. Diagnosis of coronary artery disease (CAD) was defined by the presence of at least one vessel with stenosis >50% on angiogram. In cases of symptoms compatible with coronary disease in the absence of risk factors, patients with normal exercise testing, myocardial scintigraphy, or cardiac angiotomography were considered to have no CAD.

### Electrophysiological study

Baseline measurements including AH and HV intervals were made for the definition of intranodal and intraventricular conduction abnormalities. Sinus node function was assessed by corrected sinus node recovery time and sinoatrial conduction time measurements, both obtained with rapid atrial stimulation. Right atrium, atrioventricular node, and right ventricular refractory periods were determined using standard programmed stimulation methods.

All patients underwent programmed ventricular stimulation with up to three extra stimuli and rapid ventricular pacing (up to 250ms) in the right ventricular apex and right ventricular outflow tract. Inducible sustained ventricular arrhythmias were defined as sustained ventricular tachycardia or fibrillation on the EPS.

### Statistical analysis

Continuous variables were described as mean and standard deviation or median and interquartile range, when appropriate. Categorical data were presented as relative and absolute frequencies. The difference between groups regarding inducibility and non-inducibility was analyzed by the t-test or Mann-Whitney test for continuous variables and Fisher's exact test or χ^2^ for categorical variables. A logistic regression model was used for measures of association between categories of ejection fraction (<40%, between 40 and 60%, and> 60%) and inducibility of ventricular arrhythmias on the EPS. p-values <0.05 were considered statistically significant.

## RESULTS

One hundred and forty-one charts were reviewed. We excluded 26 patients: 5 due to concomitant severe coronary artery disease, 12 due to no available Chagas serology results, and 9 due to incomplete chart information. Therefore, 115 patients were included in the analysis. Mean age was 58.4 years (SD: 10.3), 60 (52.2%) were male, 75 (65.2%) had systemic hypertension, and 104 patients (90.4%) were in either NYHA functional class I or II. Baseline characteristics are described on table 1.

**Table 1:** Baseline characteristics

Characteristics		n=115	VT/VF		
			Non-inducible 60 (52.2%)	Inducible 55 (47.8%)	p value
Age (y) - Mean (SD)		58.4 (10.3)	59.6 (10.2)	57 (10.5)	0.18
Male sex		60 (52.2)	27 (45)	33 (60)	0.10
Hyperlipidemia		42 (36.5)	24 (40)	18 (32.7)	0.41
History of syncope		60 (52.2)	29 (48.3)	31 (56.4)	0.38
Tobacco use		6 (5.2)	4 (6.7)	2 (3.6)	0.68
Prior stroke/TIA		9 (7.8)	4 (6.7)	5 (9.1)	0.73
Systemic hypertension		75 (65.2)	40 (66.7)	35 (63.6)	0.73
Diabetes mellitus		12 (10.4)	9 (15)	3 (5.5)	0.09
NYHA functional class					0.31
	I	56 (48.7)	33 (55)	23 (41.8)	
	II	2 (1.7)	23 (38.3)	25 (45.3)	
	III	48 (41.7)	4 (6.7)	5 (9.1)	
	IV	9 (7.8)	0	2 (3.6)	

VT/VF: ventricular tachycardia/ventricular fibrillation; SD: standard deviation; TIA: transitory ischemic attack; NYHA: New York Heart Association.

More than 50% of patients were on angiotensin converting enzyme inhibitor (ACEI) or angiotensin II receptor blocker (ARB), β-blockers, or amiodarone (95, 82.6%; 74, 64.3%; and 71, 61.7%, respectively). Concomitant medications are shown on table 2.

**Table 2:** Concomitant medications

Medications	n=115 (%)	VT/VF		
		Non-inducible 60 (52.2%)	Inducible 55 (47.8%)	p value
Beta-blockers	74 (64.3)	32 (53.3)	42 (76.4)	0.01
Amiodarone	71 (61.7)	34 (56.7)	37 (67.3)	0.24
ACEI/ARB	95 (82.6)	50 (83.3)	45 (81.8)	0.83
Furosemide	41 (35.7)	16 (26.7)	25 (45.5)	0.03
Statin	34 (29.6)	21 (35)	13 (23.6)	0.18
Aspirin	48 (41.7)	26 (43.3)	22 (40)	0.71
Oral anticoagulants	11 (9.6)	6 (10)	5 (9.1)	0.86
Spironolactone	45 (39.1)	17 (28.3)	28 (50.9)	0.01

VT/VF: ventricular tachycardia/ventricular fibrillation; ACEI: angiotensin converting enzyme inhibitor; ARB: angiotensin II receptor blocker.

Ninety-nine patients (86%) were in sinus rhythm and the most common conduction disturbance was left anterior fascicular block (n=62; 53.9%), followed by right bundle branch block (n=56; 48.7%). The mean ejection fraction (EF) was 45% (SD: 16.2). Table 3 summarizes the electrocardiographic, echocardiographic, and 24-hour Holter findings.

**Table 3:** Electrocardiographic, echocardiographic, and 24-hour Holter findings

Electrocardiogram findings	n=115 (%)	VT/VF		
		Non-inducible 60 (52.2%)	Inducible 55 (47.8%)	p value
Sinus rhythm	99 (86.1)	52 (86.7)	47 (85.5)	0.85
Atrial fibrillation	5 (4.3)	4 (6.7)	1 (1.8)	0.36
Paced rhythm	16 (13.9)	7 (11.7)	9 (16.4)	0.46
First degree AV block	42 (36.5)	21 (35)	21 (38.2)	0.72
Right bundle branch block	56 (48.7)	35 (58.3)	21 (38.2)	0.03
Left anterior hemiblock	62 (53.9)	36 (60)	26 (47.3)	0.17
Left bundle branch block	17 (14.8)	6 (10)	11 (20)	0.13
Low QRS voltage	9 (7.8)	5 (8.3)	4 (7.3)	1
**Echocardiogram findings** *				
Diastolic dysfunction	59 (53.2)	36 (62.1)	23 (43.4)	0.04
RV dysfunction	5 (4.5)	1 (1.7)	4 (7.5)	0.18
LV thrombus	1 (0.9)	0	1 (1.9)	0.47
LV aneurysm	6 (5.4)	3 (5.1)	3 (5.7)	1
EF (%), mean (SD)	45 (16.2)	51 (16.1)	38 (13.6)	<0.01
Left atrium (mm), mean (SD) 42.5 (7.6)	41.6 (7.8)	43.5 (7.4)	0.03
**24-hour Holter monitoring findings** **				
>30 PVC/h	64 (57.1)	32 (55.2)	32 (59.3)	0.66
NSVT	66 (58.9)	32 (55.2)	34 (63)	0.40
Sustained VT	2 (1.8)	1 (1.7)	1 (1.9)	1

* Two patients had no echocardiography available in the medical chart, one patient only had the ejection fraction described in the chart, and for one patient it was not possible to assess diastolic function due to E- and A-wave fusion.

** three patients had no 24-hour Holter monitoring result.

VT/VF: ventricular tachycardia/ventricular fibrillation; AV: atrioventricular; RV: right ventricle; LV: left ventricle; EF: ejection fraction; SD: standard deviation; PVC: premature ventricular contractions; NSVT: non-sustained ventricular tachycardia; VT: ventricular tachycardia.

### Electrophysiological study

The corrected sinus node recovery time and sinoatrial conduction time were abnormal in 8 (6.9%) and 30 (26.1%) patients, respectively. Mean corrected sinus node recovery time was 336.1ms (SD: 171.7) and mean sinoatrial conduction time was 212.8ms (SD: 82.1).

The mean AH and HV intervals were 121.4ms (SD: 56.7) and 59.2ms (SD: 16.8), respectively. Thirty-seven patients (32.2%) had abnormal atrioventricular conduction (prolonged A-H interval). Intraventricular conduction, determined by the HV interval, was abnormal in 39 (33.9%) patients (Table 4).

**Table 4:** Electrophysiological study findings

Measurement	Mean ms (SD)	Abnormal (% patients)
Corrected sinus node recovery time (normal range: 100-520ms)	336.1 (171.7)	8 (6.9)
Sinoatrial conduction time (normal range: 120-215ms)	212.8 (82.1)	30 (26.1)
AH interval (normal range: 55-120ms)	121.4 (56.7)	37 (32.2)
HV interval (normal range: 35-55ms)	59.2 (16.8)	39 (33.9)

SD: standard deviation.

Fifty-five (47.8%) patients had induced sustained ventricular arrhythmias. Most of them were monomorphic (n=46; 83.6%). Right bundle branch block was the most common morphology (n=29; 52.7%). Twenty-eight patients (50.9%) had induced sustained ventricular arrhythmias associated with symptoms or hemodynamic instability. In 51 patients (93%), the mode of reversal was described as follows: 33 (60%) required electrical cardioversion, 15 (27.3%) overdrive suppression, 1 (1.8%) terminated spontaneously, and in 2 (3.6%), the ventricular tachycardia was terminated during radiofrequency ablation. The most common site of origin of ventricular arrhythmias was the left ventricular inferoseptal wall (n=10; 18.2%), followed by the left ventricular posterobasal wall (n=6; 11%). The characteristics of induced sustained ventricular arrhythmias are described on table 5.

**Table 5:** Characteristics of sustained ventricular arrhythmias

Electrophysiological study findings		n (%)
VT/VF		55 (47.8)
	Monomorphic ventricular tachycardia	46 (83.6)
	Polymorphic ventricular tachycardia	3 (5.4)
	Ventricular flutter	3 (5.4)
	Ventricular fibrillation	3 (5.4)
Reversal of arrhythmias		
	Electrical cardioversion	33 (60)
	Overdrive	15 (27.3)
	Spontaneous	1 (1.8)
	Radiofrequency	2 (3.6)
Morphology		
	Right bundle branch block	29 (52.7)
	Left bundle branch block	15 (27.3)
	Other	11 (20)
Origin		
Left ventricle		
	Inferoseptal wall	10 (18.2)
	Posterobasal wall	6 (11)
	Inferoapical wall	2 (3.6)
	Papillary muscle	2 (3.6)
	Inferoposterior wall	1 (1.8)
	Posterolateral wall	1 (1.8)
	Inferior wall	1 (1.8)
	Anterolateral wall	1 (1.8)
	Left medial septum	1 (1.8)
	Anteroapical wall	1 (1.8)
Right ventricle		
	Right ventricular outflow tract	5 (9.1)
	Right medial septum	4 (7.3)
	Inferior/inferoapical	3 (5.4)
	Inferoseptal	1 (1.8)
	Other	2 (3.6)

VT: ventricular tachycardia, VF: ventricular fibrillation.

Among patients who developed ventricular tachycardia/ventricular fibrillation (VT/VF) on the EPS, most of them were on beta-blockers and this difference reached statistical significance (76.4% *versus* 53.3%; p=0.01). The mean ejection fraction was lower in the group with inducible VT/VF (38%, SD: 13.6 *versus* 51%, SD: 16.1; p<0.01). Patients with an EF<40% had a 1.94-fold increased risk of VT/VF seen on the EPS compared to those with an EF>60% (OR: 1.94, 95%CI: 1.12-3.38; p=0.01). Moreover, an EF>60% had a protective effect over the risk of VT/VF (OR: 0.20, 95%CI: 0.08-0.49; p<0.01). Ejection fractions between 40 and 60% had no effect on the induction of VT/VF (OR: 1.0, 95%CI 0.43-2.30; p=1). The presence of complex ventricular arrhythmias on 24-hour Holter monitoring was not associated with increased risk of inducible ventricular arrhythmias.

## DISCUSSION

The main finding of our study was that, similarly to those with both non-ischemic and ischemic dilated cardiomyopathy, patients with *Trypanosoma cruzi* infection with an ejection fraction<40% had an increased risk of inducible malignant ventricular arrhythmias. On the other hand, despite the high prevalence of frequent premature ventricular contractions, the presence of ventricular arrhythmias on Holter did not predict the inducibility of malignant arrhythmias on EPS. This most likely reflects the diffuse muscular damage in patients with CD.

Takehara et al. demonstrated the left ventricular inferolateral wall to be the most common site of origin of ventricular tachycardia in patients with CD, followed by septal and apical regions^(10)^. In our sample, the most common site of origin of ventricular arrhythmias was the left ventricular inferoseptal wall (18.2%), followed by the posterobasal region (11%). This finding reinforces the hypothesis that despite the fact that ventricular apical aneurysms are commonly seen in patients with CD (5.4% of cases in our study), most ventricular arrhythmias are not originated from or around the aneurysm. The predilection for the left ventricular wall, however, is still uncertain.

In general, an EPS is not imperative to reach a therapeutic decision in patients with sinus node disease. Pacemaker implantation is usually recommended in the presence of symptoms secondary to bradyarrhythmias, advanced atrioventricular (AV) block, and chronotropic incompetence. Programmed ventricular stimulation is usually indicated in cases of syncope of uncertain etiology^(11)^. Maia et al. and Pimenta et al. reported sinus dysfunction in 30% and 18.1% of cases of patients with chronic Chagas cardiomyopathy, respectively, which is in agreement with our findings (27% of cases)^(12,13)^.

Finally, both atrioventricular and intraventricular conduction disturbances are generally seen in clinical practice in Chagas patients. The EPS is usually indicated for patients with intraventricular block and syncope symptoms of unclear etiology^(14)^. In our study, atrioventricular node conduction abnormalities were observed in 32% of patients, and His-Purkinje system disease in 33.91%.

### Limitations

Our study has several limitations. The descriptive design, small number of patients, and information obtained based solely on medical charts are the main limitations.

## CONCLUSIONS

Chagas patients with low ejection fraction have an increased risk of inducible malignant ventricular arrhythmias. Sinus node dysfunction, atrioventricular node and His-Purkinje conduction abnormalities occur in almost one third of patients. The presence of complex ventricular arrhythmias on 24-hour Holter monitoring was not associated with increased risk of inducible ventricular arrhythmias.
